# Articular Cartilage Stem Cells Influence the Postoperative Repair of Hip Replacement by Regulating Endoplasmic Reticulum Stress in Chondrocytes via PERK Pathway

**DOI:** 10.1111/os.12644

**Published:** 2020-03-08

**Authors:** Ke Rong, Qing‐quan Xia, Xu‐hua Wu, Zhen‐yu Zhou, Xu‐jun Li, Teng Fei, Jiong Chen, Zhongyue Huang, Jiang Li, Jiang‐yi Liu, Xiao‐fan Yin

**Affiliations:** ^1^ Department of Orthopaedics Minhang Hospital, Fudan University Shanghai China

**Keywords:** Articular cartilage stem cells, Chondrocytes, Endoplasmic reticulum stress, PERK

## Abstract

**Objective:**

Endoplasmic reticulum stress (ERS) is present in chondrocytes of osteoarthritis, and the intensity of ERS is related to the degree of cartilage degeneration. *In vitro* intervention strategies can change the status of ERS and induce the inhibition of ERS‐related pathway. Therefore, this study is designed to explore the role and molecular mechanism of cartilage stem cells (ACSCs) of ERS in chondrocytes after hip replacement.

**Methods:**

Human cartilage cell lines C28/I2 were cultured as the control group. The ERS inducer was added into C28/I2 as ERS group. The third ERS + stem cells group was formed by adding cartilage stem cells into ERS group, and further transfection of si‐PERK was defined as si‐PERK + ERS + stem cells group. Cell cycle and apoptosis in the four groups were determined by flow cytometry. The protein expression of GRP78, PERK, ATF4, TMEM119, CDK4, Cyclin D, and BMP6 in chondrocytes in the four groups were investigated by western blot, and the distribution of PERK, TMEM119, and BMP6 in chondrocytes were observed by immunofluorescence assay. In addition, the transcriptional levels of Bcl2, Bax, and Caspase 3 were also determined by RT‐PCR.

**Results:**

In cell cycle assay, ERS increased the accumulation of cells in G_0_/G_1_ and G_2_/M, while cartilage stem cells weakened the effects. The apoptosis rates in control group, ERS, ERS + stem cells, si‐PERK + ERS + stem cells were 0%, 21.3%, 18.9%, and 15.9%, respectively, and the difference of apoptosis rate between the latter three groups and control group was statistically significant (*P* < 0.01). Stem cells could weaken the ERS‐induced cell apoptosis, especially reducing the number of cells in the late stage of apoptosis from 5.4% to 1.1%. The protein level of GRP78, PERK, ATF4, TMEM119, and BMP6 in the group of ERS, ERS + stem cells, and si‐PERK + ERS + stem cells were all significantly higher than those in control group, and the group of ERS + stem cells was the highest, all of the differences were significant (*P <* 0.01). However, the protein level of CDK4 and Cyclin D presented an absolutely opposite trend and the difference was still significant (*P <* 0.05). The group of si‐PERK + ERS + stem cell was lower than those in the group of ERS + stem cell but higher than those in the group of ERS (*P <* 0.05). The level of Caspase 3 in the latter three groups was significantly higher than those in the control group, and the group of ERS was the highest (*P <* 0.01). Besides, the relative level of Bcl‐2/Bax in control group was 1, but the group of ERS was about 0.5, and there was significant difference (*P < 0.01*). The ratio of Bcl‐2/Bax in the group of ERS + stem cells was more than 2 and significantly higher than those of other groups.

**Conclusion:**

ACSCs could reduce ERS‐induced chondrocyte apoptosis by PERK and Bax/Bcl‐2 signaling pathway.

## Introduction

The normal articular surface is covered with hyaline cartilage. Once the hyaline cartilage is damaged, it will cause pain, instability, and stiffness of the joints, and accelerate the degeneration of the joints, leading to the occurrence of osteoarthritis^1^.The treatment of articular cartilage injury is a quite difficult problem in the field of orthopaedics, since articular cartilage has no blood vessels, nerves, and lymphatic tissues, and its repair ability is limited. Hip osteoarthritis is one common orthopaedic disease, which is characterized by the degeneration of articular cartilage and new bone formation around the joint. Although the pathogenesis is not clear, it is widely accepted that the original lesion occurs in the apoptosis of articular cartilage chondrocytes. Chondrocytes are the only cell type in mature cartilage and are responsible for the synthesis and renewal of cartilage extracellular matrix. The balance of the decomposition and synthesis activities of chondrocytes plays an important role in maintaining the normal functions and structural integrity of articular cartilage 2, 3. Hip replacement, an artificial prosthesis replacement, can relieve great pain caused by joint problems and improve their functions and activities^4^. Currently, artificial hip replacement surgery has been recognized as one of the most successful operations in treating femoral neck fracture without causing much damage to surrounding muscles and tendons, and has been widely used in clinic.

Endoplasmic reticulum stress (ERS) is a kind of subcellular pathological state caused by various stimulations and subsequent accumulation of unfolded or misfolded proteins in the endoplasmic reticulum lumen^5^. Considering that chondrocytes are located in a special microenvironment which has neither blood vessels nor nerves, their metabolism can only be achieved through osmosis. Therefore, chondrocytes are extremely sensitive to the stimulation of various physical and chemical factors and can easily lead to ERS^6^. Moderate ERS plays an adaptive cytoprotective role, while excessive and persistent ERS can cause cell apoptosis. Previous research conducted by Takada *et al*. demonstrated that ERS of chondrocytes increased during the development of osteoarthritis (OA), resulting in increased apoptosis of chondrocytes and reduced protective response^7^. ERS is a newly discovered pathway to cause apoptosis. At present, most of the research focuses on the ERS in cells like pancreas, heart muscle, and neurons; research on the apoptosis of chondrocytes is still at the initial stage and attracts increasing interest from all over the world. PERK is an inductor protein and can be activated by auto‐phosphorylation, directly inducting the un‐foldable protein in endoplasmic reticulum lumen^8^. During endoplasmic reticulum stress, PERK can be activated by the dissociated binding immunoglobulin protein (BIP), and then specifically phosphorylated the 51 serine of eukaryotic translation initiation factor 2 alpha (EIF2α). EIF2α phosphorylation promotes the expression of activating transcription factor‐4 (ATF‐4), thus increasing the synthesis of chaperone molecules and up‐regulating the expression of genes related to amino acid metabolism, oxidative stress, and protein secretion to promote cell survival^9^. Protein TMEM119 located in endoplasmic reticulum can up‐regulate the expression of ATF‐4 to induce chondrogenic differentiation^10^.

Articular cartilage has also been proven to have a type of cartilage stem cells (ACSCs) with self‐proliferation and multi‐directional differentiation potentials. Such stem cells can spontaneously aggregate to the damaged site, which is conducive to the rapid establishment of the microenvironment for cartilage repair. ACSCs have been used in treatment for early joint injuries, inhibiting further develop of cartilage damage, as well as reducing the incidence of osteoarthritis alone or in combination with artificial replacement^11^. ERS helps the growth of osteoblasts and chondrocytes, promotes the differentiation of articular cartilage stem cells into osteoblasts, and inhibits the occurrence of apoptosis. However, under high‐intensity and long‐term stress conditions, ERS will induce the occurrence of apoptosis through multiple signaling pathways^12^. Sodium 4‐phenylbutyrate (4‐PBA), a terminal aromatic substituted fatty acid, has been recognized as the typical endoplasmic reticulum inhibitor, and various 4‐phenylbutyrate derivatives have been reported to be effective on treating neurodegenerative disease^13^.

However, the research on ERS in the field of osteoarthritis is still quite limited, and the inhibitory effect and mechanism of ACSCs on ERS are unclear. Therefore, there are two main purposes of this study: (i) to explore the role of ACSCs in ERS in chondrocytes after hip replacement by observing the changes of related genes and proteins and (ii) to elucidate the molecular mechanism of ACSCs inhibitory effects on ERS. We hope that this research can provide new ideas for alleviating ERS in chondrocytes, prevent related complications after hip replacement, and help patients reduce pain to a greater extent.

## Material and Methods

### *Cell Culture*

Human cartilage cell lines C28/I2were obtained from Chinese Academy of Sciences (Shanghai, China). Cells were grown in DMEM medium supplemented with 10% fetal bovine serum (FBS), 1 × 10^−5^/UI penicillin, and 100 mg/L streptomycin. The cell mixture was cultured at 37°C in a humidified 5% CO_2_ incubator.

### *Transfection of siRNAs*

We bought human PERK siRNAs from Santa Cruz Biotechnology. Cells were put in plates containing Dulbecco's modified Eagle medium and 10% FBS. Then, plates were placed in incubator with 5% CO_2_ andcultured for 72 h after transfection of 80 nmol/L PERK siRNAs. The transfection was performed by Lipofectamine 2000 Transfection Reagent.

### *Establishment of Models*

Hypoxia injury model was established by using hypoxia bag *in vitro* to create hypoxia environment. Four groups were arranged in this study. A group contained normal human cartilage cells C28/I2 and worked as the control. Moderate endoplasmic reticulum stress was induced in human cartilage cells C28/I2 by exposure to tunicamycin, and these cells were further divided into three groups including B, C, and D group. Group B contained only C28/I2 cells that were subject to ERS. Group C contained the co‐culture system of cartilage stem cells and chondrocyte ERS model. The constructed si‐PERK interfering plasmid was transfected into chondrocyte ERS model to form D group together with cartilage stem cells.

### *Flow Cytometry for Cell Apoptosis*

The cells, before and after transfection and at logarithmic growth stage, were digested into a single‐cell suspension and centrifuged. The supernatant was discarded and cells were stained using Annexin V/propyl iodide. Apoptosis was detected by flow cytometry according to the manufacturer's instructions.

### *Western Blot Analysis for Protein Expression*

Cells were collected for protein expression determination. After digestion with 0.25% trypsin, the cells were washed with precooled PBS three times. Protein lysates were added to lyse the cells, and the total proteins in the cells were extracted. Equivalent protein samples were taken and subjected to SDS‐PAGE electrophoresis. Separated proteins on the gel were transferred to cellulose nitrate membrane. Reaction was conducted in 5% skim milk powder sealant for 2 h. Primary anti‐GRP78, PERK, ATF4, TMEM119, CDK4, CyclinD, and BMP6 were added and incubated at 4°C overnight. Secondary antibodies were added and incubated at room temperature for 2 h. The mixture was then washed with phosphate buffered solution (PBST) three times. ECL was used for chemiluminescence. Photos were taken and gray value of each strip was analyzed by the Image J Image analysis system.

### *RT‐PCR for mRNA Expression of Bcl2, Bax, and Caspase 3*

Total RNA was extracted from cells by Trizol method and cDNA was synthesized according to the instructions of reverse transcription kit. PCR amplification was performed as follows: 95°C 5 min, 95°C 20 s, 60°C 30 s, and 72°C 20 s for 40 cycles. U6 was taken as a control. The primers were listed in the following Table 1

**Table 1:** List of primers used in RT‐PCR

Gene name	Primer forward	Primer reverse
Bcl2	GGTGGGGTCATGTGTGTGG	CGGTTCAGGTACTCAGTCATCC
Bax	CCCGAGAGGTCTTTTTCCGAG	CCAGCCCATGATGGTTCTGAT
Caspase 3	TTTCTGCCTACAGGGTCATGC	GCTGCTTCTCTCTTTGCTGAA

### *Immunofluorescence Assay for the Distribution of Proteins*

The cultured C28/I2 cells were washed three times with PBS and blockaded with 10% rabbit serum at 37°C for 30 min. Then, the cells were incubated with the primary antibodies for ERK, TMEM119, or BMP6 overnight at 4°C (1: 100). Afterwards, the cells were incubated with Alexa Fluor 488 Goat Anti‐Rabbit IgG (1: 100) for 30 min at 37°C and washed with PBS. Observation and photography were performed with an Olympus multifunction microscope (Olympus BX51, Beijing, China).

### *Statistical Analysis*

Quantitative data were analyzed using SPSS 20.0 (IBM SPSS Statistics, IBM Corp, Somers, NY, USA) and expressed as the mean ± SD. Statistical differences between groups were compared using ANOVA and two‐tailed *t*‐tests. *P <* 0.05 was considered statistically significant.

## Results

### *Cell Cycle of Chondrocytes after Treatment with ERS or si‐PERK*

**Figure 1:**
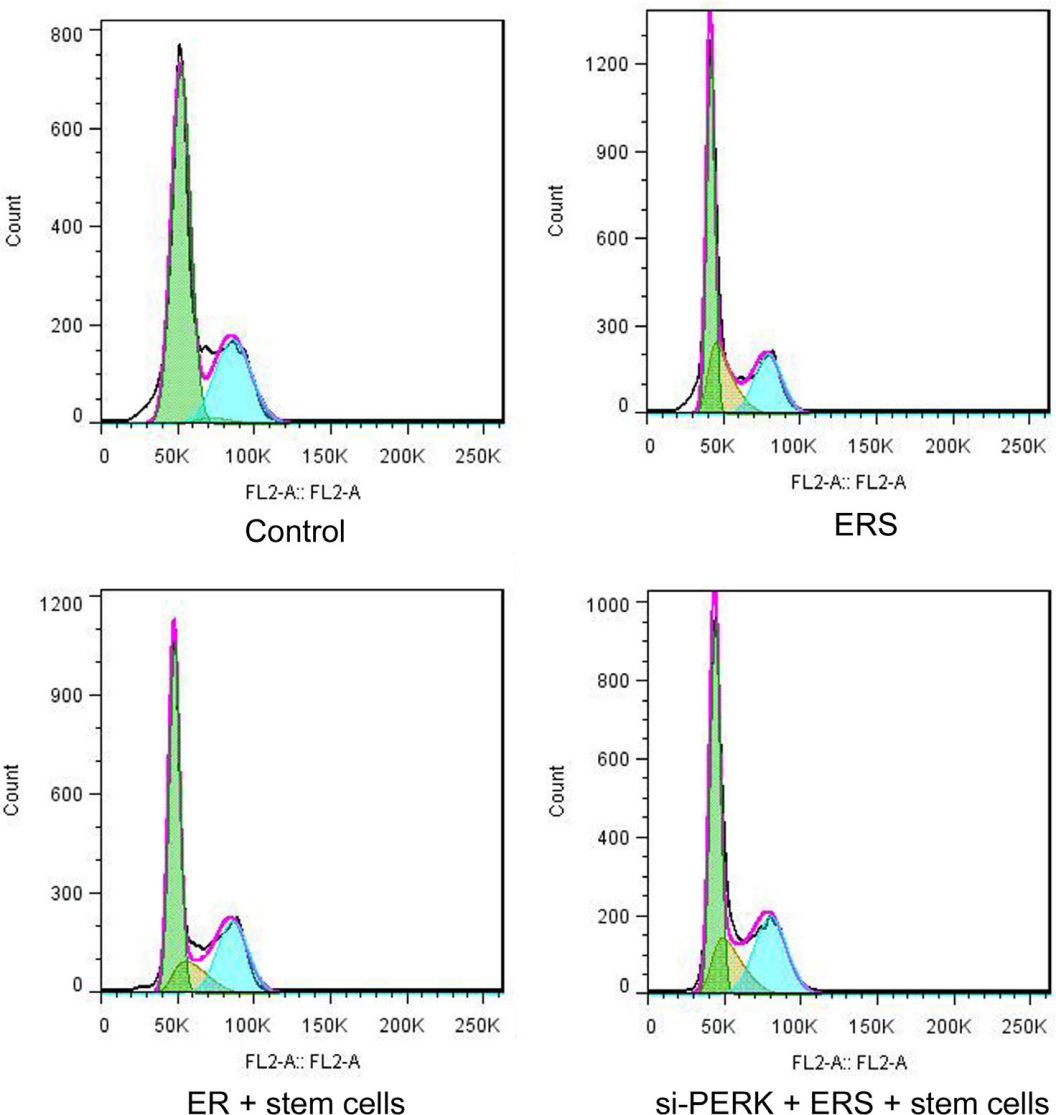
ERS could regulate the cell cycle of stem cells in chondrocytes. Chondrocyte cycle in the control (A), ERS (B), ERS + stem cell (C) and si‐PERK + ERS + stem cell group (D). The peak value of G_0_/G_1_ and G_2_/M phase in si‐PERK + ERS + stem cell group was lower than that in ERS + stem cell group, while higher than ERS group.

The cell cycle results in the control, ERS, ERS + stem cell and si‐PERK + ERS + stem cells groups were showed in Fig. 1. As shown, the peak values of both G_0_/G_1_ and G_2_/M phases of chondrocytes in ERS group were lower than the corresponding phases in control group. The peak values of G_0_/G_1_ and G_2_/M phases in ERS + stem cell group were lower than those in control group, while higher than those phases in ERS group, suggesting that cartilage stem cells alleviated the effects on cell cycle caused by ERS. Besides, the peak values of G_0_/G_1_ and G_2_/M phases in si‐PERK + ERS + stem cell group were lower than those in ERS + stem cell group, while higher than ERS group. The silence of PERK pathway reduced the inhibitory effects of stem cells on endoplasmic reticulum stress. These results indicated that ERS could regulate the cell cycle of stem cells in chondrocytes.

### *Cartilage Stem Cell Weakens ERS‐induced Cell Apoptosis*

**Figure 2:**
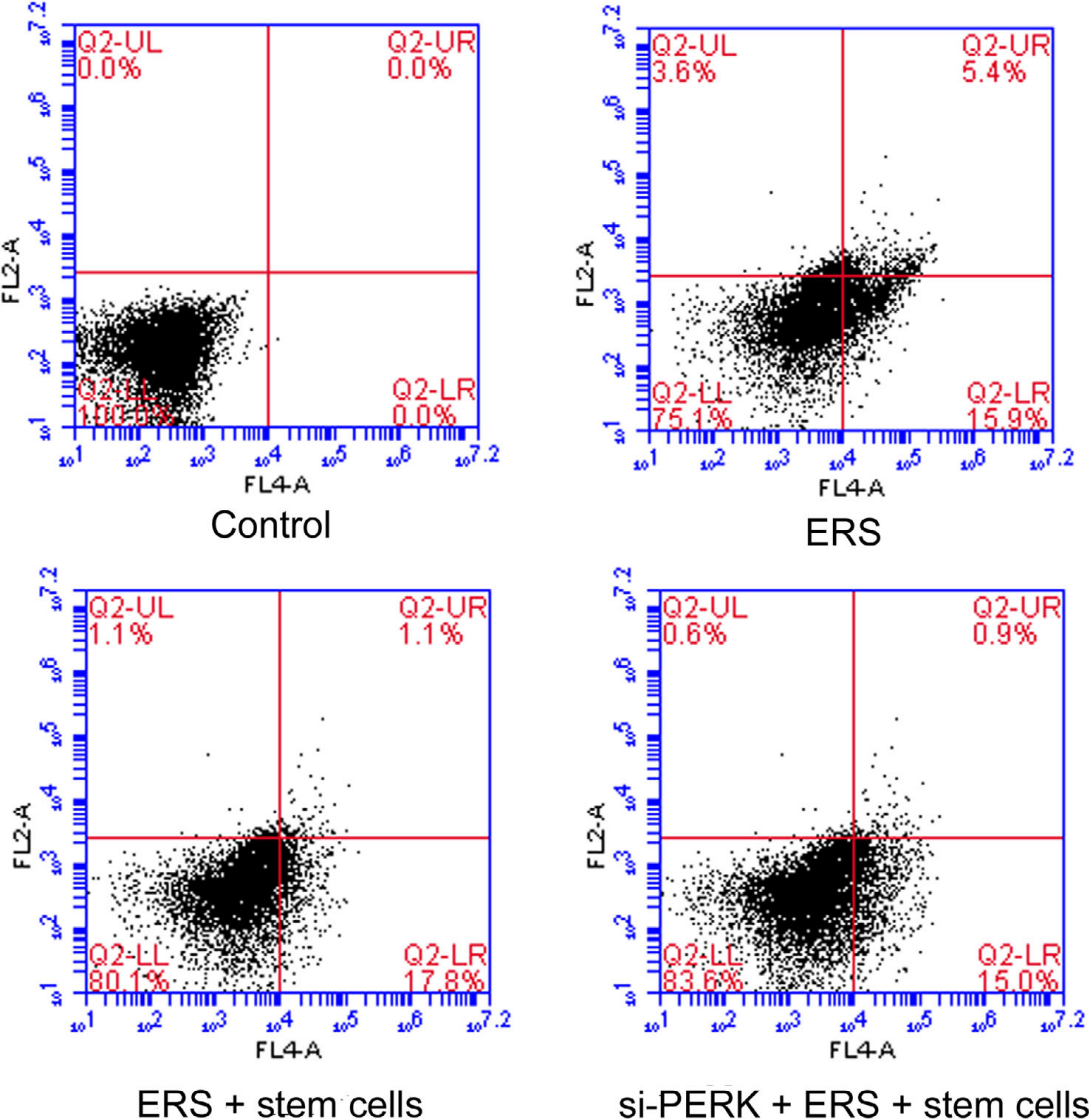
The ERS induced cell apoptosis was decreased by cartilage stem cells. Chondrocyte apoptosis percent in the control (A), ERS (B), ERS + stem cell (C) and si‐PERK + ERS + stem cell group (D) determined by flow cytometry.

Phosphatidylserine (PS) is normally located on the inner side of the cell membrane, but, in the early stage of apoptosis, PS flips from the inner side of the membrane to the membrane surface. Annexin V can bind to PS with high affinity and cells marked with Annexin V are considered as apoptotic cells. PI can penetrate into cells with damaged membrane. Therefore, the total proportion of Annexin V/PI‐ quadrant and Annexin V/PI quadrant are considered as apoptotic cells. The apoptosis rates of chondrocytes in the control, ERS, ERS + stem cell and si‐PERK + ERS + stem cell groups were showed in Fig. 2. As shown, the apoptosis rates in the latter three groups are all higher than that in the control group (0%), and the apoptosis rate in ERS group (21.3%) was the highest, followed by that in ERS + stem cells group (18.9%) (*P < 0.01*).

Stem cells could weaken the ERS‐induced cell apoptosis, especially reducing the number of cells in the late stage of apoptosis from 5.4% to 1.1%. Besides, the apoptosis rate in si‐PERK + ERS + stem cells group was 15.9%. Elimination of the PERK gene could reduce the proportion of apoptotic cells in chondrocytes, indicating that the PERK pathway was the main factor inducing cell apoptosis during endoplasmic reticulum stress. These results demonstrated that the ERS‐induced cell apoptosis was decreased by cartilage stem cells.

### *Cartilage Stem Cell Affects the Expression of Transcription Factors such as GRP78, PERK, ATF4, TMEM119, CDK4, Cyclin D, and BMP6*

**Figure 3:**
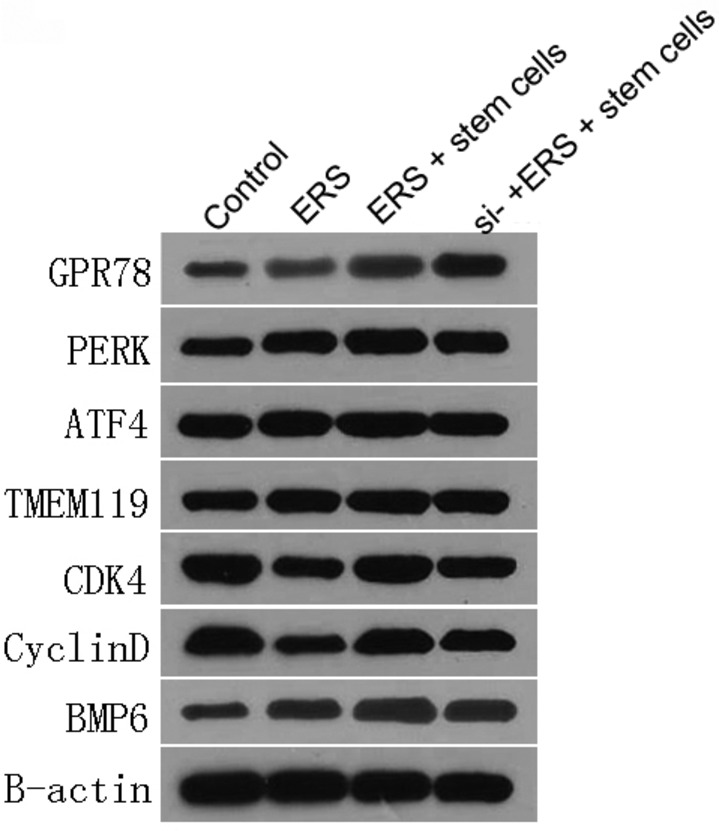
Cartilage stem cells mediated the expression of various transcription factors. The protein expression values of GRP78, PERK, ATF4, TMEM119, CDK4, Cyclin D and BMP6 in chondrocytes in the control, ERS, ERS + stem cell and si‐PERK + ERS + stem cell group by western blot assay.

Western blot assay was used to determine the protein expression values of GRP78, PERK, ATF4, TMEM119, CDK4, Cyclin D, and BMP6 in chondrocytes in the four groups. As Fig. 3 showed, the protein expression values of GRP78, PERK, ATF4, TMEM119, and BMP6 in ERS, ERS + stem cells, and si‐PERK + ERS + stem cells groups were all significantly higher than those in control group, and the protein expression in ERS + stem cells was the highest (*P <* 0.01). The expression values in si‐PERK + ERS + stem cell group was higher than those in ERS group, but lower than those in ERS + stem cells group (*P <* 0.01). The protein expression values of CDK4 and Cyclin D in ERS, ERS + stem cell, and si‐PERK + ERS + stem cell groups were all markedly lower than those in control group, and the values in ERS group were lowest among them (*P <* 0.05). The values in si‐PERK + ERS + stem cell group were lower than those in ERS + stem cell group but higher than those in ERS group (*P <* 0.05). Based on these results mentioned above, we proposed that cartilage stem cells mediated the expression of various transcription factors.

### *Immunofluorescence Results of the Distribution of PERK, TMEM119, and BMP6 in Chondrocytes*

**Figure 4:**
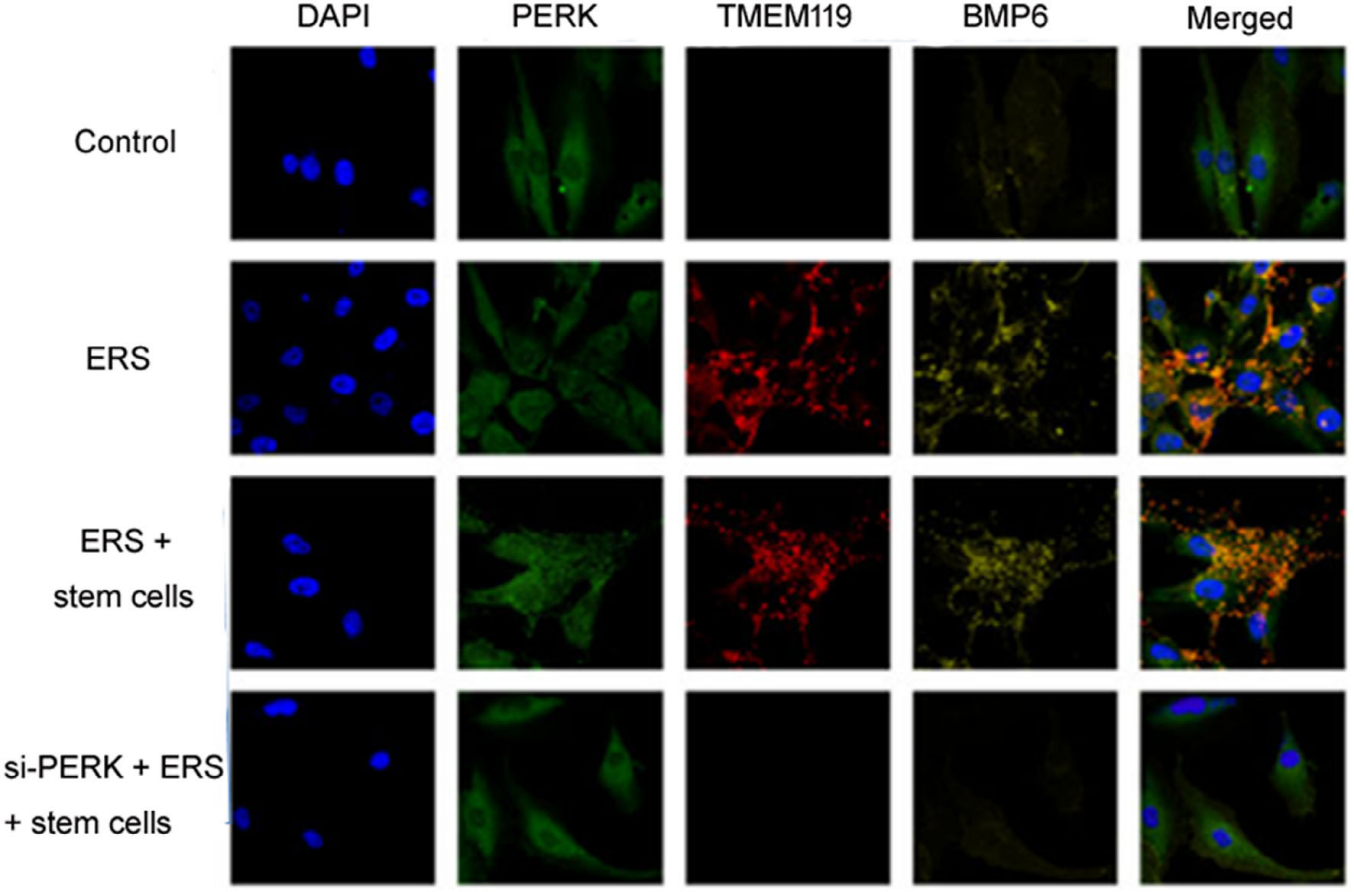
ERS could affect the distribution of PERK, TMEM119 and BMP6 in chondrocytes. The location and expression level of PERK, TMEM119 and BMP6 in chondrocytes determined by immunofluorescence assay.

Immunofluorescence assay was applied to study the location of PERK, TMEM119, and BMP6 in chondrocytes. As shown in Fig. 4, the protein BMP6 was located in the membrane of chondrocyte. Protein PERK and TMEM119 were located in endoplasmic reticulum of chondrocyte. The relative transcript levels of PERK, TMEM119, and BMP6 is shown as highest in ERS + stem cells group while lowest in control group. The relative transcript levels in si‐PERK + ERS + stem cell group were higher than those in ERS group. Therefore, we concluded from these results that ERS could affect the distribution of PERK, TMEM119, and BMP6 in chondrocytes.

### *Cartilage Stem Cell AffectsBcl2, Bax, and Caspase 3 Levels*

**Figure 5:**
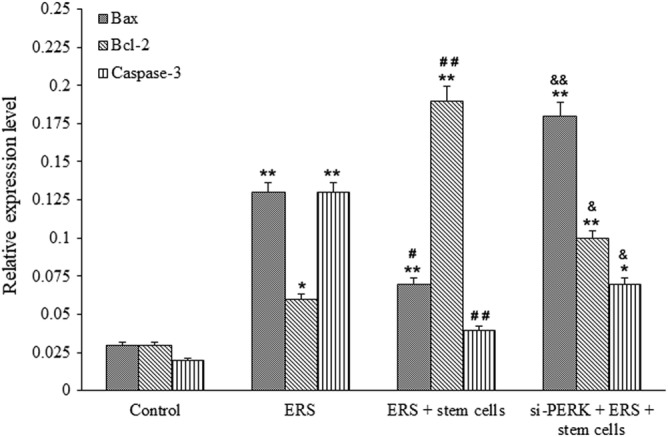
Cartilage stem cells could influence the expression of Bcl2, Bax and caspase 3 after treated with ERS. The relative expression levels of Bcl2, Bax and Caspase 3 in chondrocytes in the control, ERS, ERS + stem cell and si‐PERK + ERS + stem cell group determined by RT‐PCR. **P < 0.01 or *P < 0.05, compared with the control group; ^##^ *P <* 0.01 or ^#^ *P <* 0.05, compared with the ERS group; ^&&^ *P <* 0.01 or ^&^ *P <* 0.05, compared with the ERS group.

The relative expression levels of Bcl2, Bax, and Caspase 3 in the control, ERS, ERS + stem cell, and si‐PERK + ERS + stem cell groups were showed in Fig. 5. Caspase 3 and Bax (a Bcl‐2 family member) are two common pro‐apoptotic proteins, and Bcl‐2/Bax ratio determines the trend of apoptosis. Increased ratio of Bcl‐2/Bax inhibits cell apoptosis and decreased ratio promotes cell apoptosis. As shown, the relative expression levels of Caspase 3 in the other three groups were all significantly higher than those in the control group, and ERS group showed the highest value (*P <* 0.01). The relative expression of Caspase 3 in si‐PERK + ERS + stem cells group was higher than that in ERS + stem cells group (*P <* 0.01). Besides, the relative expression level of Bcl‐2/Bax in control group was 1, the levels in ERS was about 0.5, indicating ERS‐induced apoptosis (*P <* 0.01). Bcl‐2/Bax expression ratio in ERS + stem cells group was more than 2, and the increased value suggested that apoptosis caused by ERS was weakened. Transfection of si‐PERK reduced the effect of stem cells, but the Bcl‐2/Bax expression ratio was still higher than that in ERS group. These results showed that cartilage stem cells could influence the expression of Bcl2, Bax, and Caspase 3 after treated with ERS.

## Discussion

### *Brief Introduction of ERS*

Endoplasmic reticulum (ER) is a fine membrane system in cells, which plays an important role in cell growth, survival, and function^14^. ER mainly affects the physiological processes such as protein secretion, lipid synthesis, and calcium ion balance^15^. A variety of stimuli in the internal and external environment, such as tissue hypoxia, REDOX injury, high‐fat diet, hypoglycemia, protein inclusion bodies, and viral infections, can cause the accumulation of unfolded proteins in the endoplasmic reticulum, thus leading to intracellular stress response, and this process is called endoplasmic reticulum stress (ERS)^16^. In recent years, ERS was reported to be associated with many genetic and non‐genetic diseases in humans, including neurodegenerative diseases, multiple sclerosis, metabolic diseases, cancer, and inflammatory diseases. However, there are few reports on the effects of ERS on chondrocytes.

### *Potential Function of ERS in Chondrocytes*

Chondrocytes are the only cellular components found in normal articular cartilage. Chondrocytes are in a special microenvironment without vascular, nerve and lymphatic distribution, and various physical and chemical factors are likely to stimulate ERS^17^. ERS can directly or indirectly induce chondrocyte apoptosis by reducing the expression of type II collagen and proteoglycan, the main components of cartilage matrix^18^.

Cartilage stem cells (ACSCs) can be grown into mature chondrocytes. ERS regulates cell apoptosis by activating the unfoldable protein response (UPR). UPR includes three signaling pathways, namely PERK, ATF6, and IRE1. PERK is endoplasmic reticulum type I transmembrane protein. It is activated by autodimerization and phosphorylation after dissociation. Activated PERK phosphorylates translation initiation factor 2α (EIF2α), which selectively facilitates the translation of ATF‐4, thus the ERS stress response was activated and the apoptosis process was initiated. In our study, the apoptosis rate in ERS group was significantly higher than the control group. Addition of ACSCs decreased the cell apoptosis induced by ERS, while further transfection of si‐PERK weakened the inhibitory role of ACSCs on ERS‐induced cell apoptosis. Moreover, the expressions of PERK and ATF‐4 were found significantly up‐regulated by ERS.

Co‐culture of ACSCs with ERS cells promoted the protein expression of PERK and ATF‐4, while further transfection of si‐PERK abolished the promoting role. GRP78, also referred to as BIP, is a central regulator of endoplasmic reticulum function due to its roles in protein folding and assembly, targeting misfolded protein for degradation, ER Ca^2+^‐binding, and controlling the activation of trans‐membrane ERS sensors^19^. TMEM119 is a member of the transmembrane proteins family, which is abnormally expressed in human cancers and associated with tumorigenesis^20^. Cyclin D‐CDK4 complexes have been characterized as growth factor‐responsive cell cycle regulators. BMP6 is a transforming growth factor beta superfamily member produced by mammalian oocytes as well as other cell types^21^. It is an important regulator of cell growth, differentiation, and apoptosis in various types of tumor (Reduced BMP6 expression by DNA methylation contributes to EMT and drug resistance in breast cancer cells. BMP‐6 is an autocrine stimulator of chondrocyte differentiation^22^). Several investigators reported that BMP‐6 plays a role in chondrocyte differentiation.

Our study showed ERS and ACSCs up‐regulated the expression of GRP78, PERK, ATF4, TMEM119, and BMP6, which knockdown of PERK weakens the up‐regulation role. ERS decreased the level of CDK4 and CyclinD, while the addition of ACSCs increased the expression of these genes. Further knockdown of PERK weakened the increased role of ACSCs. Bcl‐2 family is an important regulatory signal molecule of apoptosis signal transduction and Caspase is the terminal molecule of apoptosis. Our study showed that ERS induction could increase the expression levels of Bax/Bcl‐2 and Caspase‐3. Adding of ACSCs alleviated the apoptosis levels induced by ERS, while further transfection of si‐PERK abolished the decreased role of ACSCs. From above results, we speculated ACSCs can reduce ERS‐induced chondrocyte apoptosis by PERK and Bax/Bcl‐2 signaling pathway.

### *Summary of Results*

There are still limitations in this study. We know that the gene transcription and protein expression are not necessarily the same, so it is better to test the Bcl‐2, Bax, and Caspase 3 protein expressions by using western‐blot assay. Besides, we found that stem cells could up‐regulate PERK protein expression and inhibit cell apoptosis. However, activation of PERK pathway could trigger apoptosis by ERS induction. The complex relationship among stem cell, ERS, and PERK needs more investigation. Factors like the concentration of stem cells and degree of stress may have pivotal effects on the regulation of ERS and underlying mechanism of stem cells in chondrocytes. Moreover, the study is only conducted at cell level; animal and human models are necessary for further verification.
